# The sentinel node concept in prostate cancer: Present reality and future prospects

**DOI:** 10.4103/0970-1591.44246

**Published:** 2008

**Authors:** M. Egawa, M. Fukuda, H. Takashima, T. Misaki, K. Kinuya, S. Terahata

**Affiliations:** Department of Urology, Tonami General Hospital, Tonami, Japan; 1 Department of Nuclear Medicine, Tonami General Hospital, Tonami, Japan; 2 Department of Clinical Pathology, Tonami General Hospital, Tonami, Japan

**Keywords:** Lymph node dissection, prostatic neoplasm, sentinel node

## Abstract

A sentinel node (SN) is defined as the first site where cancer cells are carried by lymph flow from a tumor. If this definition (SN concept) correctly reflects the clinical reality, intraoperative SN biopsy would facilitate precise nodal staging. In malignant melanoma, a prolonged survival has been evidenced by a large-scale randomized controlled study. On the contrary, research on SN concept in deeply located cancers including prostate cancer, is still investigative, and no concrete data from clinical trials are yet available. Since 1993, several investigators have demonstrated that the SN concept could be applied in prostate cancer patients as well with high accuracy. Although promising and technically feasible in pre-clinical settings, many hurdles remain to be cleared before clinical application can be recommended. This review addresses the current status and related issues of the SN concept in prostate cancer, and discusses the future directions.

## INTRODUCTION

When resecting malignant foci surgeons must always take into account the presence of invisible micrometastases so as to achieve the best possible prophylactic and systematic lymph node (LN) dissection. However, the more extensive the LN dissection the greater becomes the degree of surgical invasiveness, resulting in increasing organ functional impairment postoperatively. It would be ideal to be able to differentiate beforehand cases requiring and not requiring LN dissection, although the precision of currently available diagnostic imaging modalities is not yet sufficient for this purpose.[1] As one approach to help overcome this problem the clinical application of the sentinel node (SN) concept has been attracting increasing attention. This concept was first proposed in the early 1990s,[2] and as is well known, has been widely applied internationally especially for breast cancer[3] and malignant melanoma.[4] On the other hand, in the case of malignancies situated deep within the body, like prostate cancer, the applicability of the SN concept is still in the investigative stage with no firm conclusion yet reached as to its validity.[5,6]

SN is defined as the first LN to receive lymph flow from a tumor. The SN concept is based on the assumption that metastases would first develop in this SN. In other words, if no metastasis is present in this SN, metastases can be judged to be absent in other LN, and gratuitous LN dissection can be omitted thereby reducing surgical invasiveness. Conversely, when metastasis is present in the SN, the LN dissection can be extended facilitating a switch to a more radical surgical method. In this way, SN navigation surgery (SNNS) can be used to determine the indications for radical surgery according to the presence/absence of SN metastases and decide the extent of the LN dissection required, but in prostate cancer this is still limited to the basic science or clinical research stage and has in general not yet been applied clinically. This review first discusses the various issues related to LN dissection in prostate cancer, then, notes the basic research results validating the SN concept, and finally outlines the results of recent clinical research and future prospects.

## VARIOUS ISSUES RELATED TO LN DISSECTION IN PROSTATE CANCER

In contrast to numerous other malignancies, in prostate cancer various issues remain to be resolved regarding LN dissection.

### The extent of dissection

Usually, in any kind of malignancy the extent of LN dissection is determined by the state of the primary lesion (or regardless of its state), with the presence/absence of LN metastases diagnosed based on the pathological results of the dissected LN. If this definition is not clarified comparisons of the results of studies on LN metastases conducted by different surgeons at different institutions will be problematic. When commencing research on LN in prostate cancer this is the first issue that has to be confronted. Even if a study specifies ‘limited dissection’, ‘standard dissection’, and ‘extended dissection’, in practice in many cases it will be impossible to know the actual extent of the dissection. Moreover, in some papers the extent of the dissection is not specified at all.

### The staging significance of lymph node dissection

Since metastatic LNs of prostate cancer are not markedly enlarged and are usually microscopic in nature, the usefulness of diagnostic imaging in detecting them is low. Recently, modalities such as Positron Emission Tomography-Computerized Tomography (PET-CT) are being used, but their diagnostic rate of clinically localized prostate cancer is unfavorable.[7] Accordingly, although extending the range of the dissection would theoretically facilitate a more accurate diagnosis of LN metastases, the burden of complications such as edema of the lower extremities and lymphorrhea might be increased.[8,9] On the other hand, if according to nomograms such as Partin Tables[10] and Kattan nomogram[11] the probability of LN metastases is below a given level, omission of LN dissection at the time of radical prostatectomy (RPx) may also be a reasonable option at present.[12]

However, in studies in which extended LN dissection was performed for clinically localized cancer, metastases limited to beyond the range of standard dissection were found at high rates, namely 35%[13] and 19%[14] of metastasis-positive cases. Moreover, discrepancies in the histological findings are occasionally noted between biopsy specimens and totally resected specimens, with underestimation of the pathological stage becoming a problem when nomograms are used.[15]

### Therapeutic significance of lymph node dissection

Bhatta-Dar *et al*., performed RPx in 336 patients with low-risk prostate cancer (with dissection 140 cases/without dissection 196 cases) and retrospectively noted no difference in the six-year biochemical recurrence-free survival rate between the two groups.[16] In the study of Salomon *et al*., in which 43 cases receiving perineal RPx without dissection and 25 cases undergoing retropubic RPx with dissection were compared, no difference in the five-year recurrence rate was found either, although the numbers were small.[17]

On the other hand, Bader *et al*., who performed extended dissection in 367 patients with prostate cancer, reported that in the 40% of cases with only a single pelvic LN metastasis no biochemical recurrence was detected during a 45-month follow-up.[18] Similarly, Han *et al*. and Daneshmand *et al*., reported that in patients with LN metastases biochemical non-recurrence rates of 15% and 21% at ≥10 years respectively were observed.[19,20] In this way, it is clear that no consensus has yet been reached with respect to the therapeutic significance of pelvic LN dissection in prostate cancer.

#### Significance of SN in prostate cancer

In prostate cancer, not only the significance of the dissection itself but also the optimal method to achieve it have yet to be resolved, especially since it is difficult to accurately grasp the presence/absence of metastases. For example, even if the nomograms are used, the original data reflect the pathological results of only some of the intrapelvic LN, precluding the making of accurate predictions. Also, it is self-evident that the more limited the extent of the dissection is the less accurate will the diagnosis of metastases be.

In this context, it is very important to validate the SN concept as verified by extended dissection. If like in breast cancer and malignant melanoma the SN concept is validated, accurate information about the LN will be obtainable by biopsy of the SN alone. Moreover, based on the information on the state of LN metastases provided by the SN biopsy, SNNS can be performed to determine the appropriateness of radical treatment. In cases negative for LN metastases in which a long-term course can be anticipated, prostatectomy or radiotherapy can be applied, whereas in cases found to be metastasis-positive conservative treatment such as hormone therapy can be resorted to so as to spare the patient gratuitous invasiveness.

#### Validation of the SN concept in prostate cancer

Since the 1999 report of Wawroschek *et al*., several groups have published papers regarding validation of the SN concept.[5,21–25] The largest scale study was that of Wawroschek *et al*., in which the results of 1055 cases were analyzed.[6] Of these, LN metastases were found in 207 cases (19.6%), with false negative results (non-SN metastases found in the absence of SN metastases) noted in only 2/207 cases (1%). In 131/207 cases (63.3%), metastases were found beyond the range of routine dissection (external iliac LN + obturator LN). Other groups including our own have obtained similar results in smaller scale investigations [Table 1]. Techniques to validate the SN concept are outlined below.

**Table 1:** Contemporary series of lymphatic mapping and SN validation studies

Series	Approach	Tracer	Extent of dissection	No. of SN	SN identification rate	Complication	False negative
Brenot-Rossi *et al*. (n= 100, 2008)	open	nanocolloid	extended	mean 3	69.4% in 60 MBq tracer 92.9% in 200 MBq tracer	not stated	2%
Weckermann *et al*. (n= 1055, 2007)	open	nanocolloid	SN only ˜ extended depending on risk factors	mean 7	97.2%	not stated	0.2%
Fukuda *et al*. (n=42, 2007)	open	phytate	extended	mean 4.9	97.6%	none	2.4%
Haker *et al*. (n=20, 2006)	laparoscopic	human albumin	extended	not stated	94.3%	1 neuropraxy 1 deep vein thrombosis 1 lumphocele	5%
Corvin *et al*. (n=28, 2006)	laparoscopic	nanocolloid	standard	mean 2.1	92.9%	none	0%

### Tracers and their method of administration

Tracers used in this context include Technetium (Tc)-labeled colloid, which is a radioisotope, and dyes. Dyes are seldom used alone and in most cases are used in combination with radioisotopes. Since dyes are taken up by lymph flow almost immediately after injection into the tissues, intraoperative observation under direct vision is required. When LNs are destroyed by surgical manipulations, the dye will flow out of the LN making accurate identification of the SN impossible. In surface cancers such as breast cancer and malignant melanoma this dye method is extremely useful, whereas in prostate cancer identification of the SN is extremely difficult using this dye. This is because when the peritoneum is not detached from the pelvic floor LN stained with the dye cannot be verified visually. To achieve this, the peritoneum should be detached from the pelvic floor beforehand, although it is difficult to completely avoid damaging the LNs during this procedure. For this reason, use of radioisotopes is indicated for the identification of SN in prostate cancer.

Colloid particle diameter is an important factor determining the speed at which the tracer passes through the LN and the degree of entrapment of the colloid within the LN.[26] According to the type of colloid used as well as the timing of SN detection after tracer administration, the number of SN that can be pointed out will vary, and thus the number and distribution of SN will differ according to the type of colloid used and the individual investigator. Tracers are usually injected transrectally to the prostate, with one injection each to the left and right lobes in most cases. The administered dose also differs according to the individual investigator with the total dose usually in the range of about 0.2 cc to 3 cc. Our group injects tracer (^99m^Tc-phytate: 40 MBq/0.1 ml/site) under transrectal ultrasonography 5-6 h prior to surgery into the right and left peripheral zones of the prostate.

### Identification of SN

Here, we introduce our SN identification method using ^99m^Tc-phytate.[27] One hundred and eighty minutes after tracer injection lymphoscintigraphy is performed, and LN in which radioisotopes have been taken up, namely SN, are depicted on the film. The number of SN will vary according to the time elapsing from the time of tracer injection to imaging, with the optimal time until imaging differing according to the type of colloid used.

**Figure 1:**
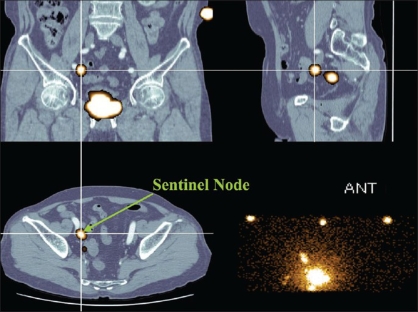
An SN is clearly indicated at the right external iliac region on CT-SPECT fusion image

Lymphoscintigrams can be displayed as planar images or single photon emission computed tomographs (SPECT), but SPECT imaging is preferable to discern the three-dimensional location of SN. Moreover if CT-SPECT fusion images are prepared the anatomical position of SN will be even better appreciated [Figure 1]. Three sites including the umbilicus are marked on CT and SPECT, and a fusion image is obtained by overlapping the marked sites of the two images on a computer.[28,29] At the time of intraoperative probing the information provided by this fusion image facilitates more accurate intraoperative detection of SN, and reduces the operating time for fusion-image supported SN resection.[30]

Since validation of the SN concept requires knowledge of the state of not only SN but also non-SN metastases, backup extended dissection is usually performed after SN biopsy. Jeschke *et al*., perform SN biopsy and extended dissection laparoscopically, and to avoid the shine-through phenomenon, use a gamma probe that can detect gamma rays from 90 degrees laterally.[31] Although a strict learning curve exists, laparoscopic extended pelvic lymphadenectomy is worth performing for validation of the SN concept because this procedure yields an equivalent number of resected LNs with less morbidity.[32] Since in most other reports, validation has been performed under laparotomy, we introduce the method adopted by us here.

**Figure 2:**
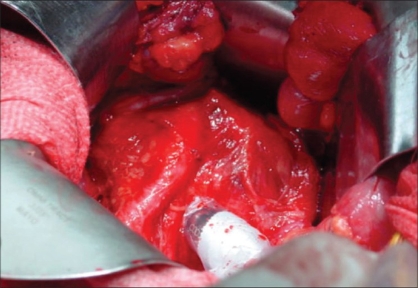
A handy type gamma probe is used to detect SNs (*in-vivo* probing)

**Figure 3:**
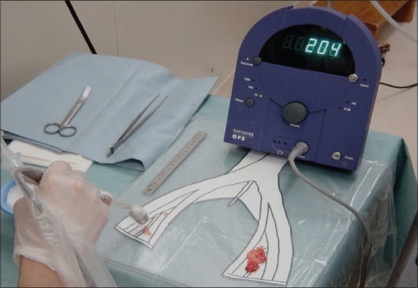
On a back table the LNs are mapped separately, and the SN more accurately identified (*ex-vivo* probing)

In short, through a lower abdominal midline incision the LN groups below the aortic bifurcation are located, and the SN is identified with a gamma probe. The gamma probe used intraoperatively is usually a handy type weighing about 200-300 g, which measures gamma rays released from the radionuclide at the tip detector portion [Figure 2]. LN identified by the gamma probe as SN are sampled, after which backup extended dissection is conducted, with the right and left common iliac vessels, external iliac vessels, internal iliac vessels, obturator fossa and presacral LN groups all resected separately. On a back table the LNs are mapped separately, and the SN more accurately identified [Figure 3]. The prostate injected with tracer emits high levels of radioactivity, which interfere with the detection of weak gamma rays from the SN, with this referred to as the shine-through phenomenon. To prevent the occurrence of this phenomenon, the prostate is covered with a lead shield.

### Pathologic diagnosis

The pathologic method used to diagnose resected LN differs with the individual investigator. In Wawroschek *et al*.'s method, the resected SN is cut into 2-mm slices, with five serial sections prepared from each slice. One of these slices is subjected to immunohistochemical staining using anti-cytokeratin antibody, and the remaining slices to routine hematoxylin and eosin staining.[33] To achieve a more detailed diagnosis of metastases we prepare serial sections from slices cut at 250 micrometer thickness, and like Wawroschek *et al*., perform immunohistochemical and H and E staining. Although the definition of micrometastases and their biological significance remain controversial, it is recognized that the diagnostic efficacy rate can be enhanced by 10-30% by adding immunohistochemical to H and E staining.[34]

## RESULTS

As noted above, the results of the largest scale study conducted by Wawroschek *et al*., supported the validation of the SN concept in prostate cancer. Since in their study false negative results were limited to only 2/207cases (1%), the diagnosis of metastases based on SN biopsy can be concluded to be a highly specific investigative method. On the other hand, excluding the two false negative cases, metastases were found in non-SN as well in 42 of 205 cases (20.5%). These findings suggest that SN biopsy alone may be of little therapeutic significance. Our data support the results of Wawroschek *et al*., and in the approach adopted by us SN biopsy positive cases undergo extended dissection and all metastatic LN are resected.

Hitherto it has been widely recognized that neoadjuvant hormone therapy (NHT) decreases the accuracy of the pathologic diagnosis of RPx specimens. Here we introduce the results obtained from our SN research based on extended dissection and investigation of micrometastases that suggest that NHT similarly interferes with the accuracy of the diagnosis of LN metastases. Extended dissection and LN mapping were conducted in 42 patients with prostate cancer (mean age 67.8 years, mean PSA 25.1 ng/ml) without clinically evident metastases. The mean number of resected LN was 26.3. In the group in which NHT was not administered (n = 15), four cases were diagnosed as metastasis positive by pathologic examination of routinely H and E-stained largest cut sections of resected LN, with this number not increasing when micrometastasis diagnosis was added. On the other hand, in the group in which NHT was given (n = 27), four cases were diagnosed as metastasis positive by routine pathologic examination (14.8%), with this number rising by five to a total of nine (33.3%) when micrometastasis diagnosis was added [Table 2]. In other words, these results imply that had NHT been given to the former group, micrometastases would have been overlooked in five cases. Thus, despite the fact that the definition of micrometastasis is unclear and their influence on prognosis is unknown, NHT was shown to decrease the diagnostic efficacy rate of LN metastases.

**Table 2:** LN metastasis by method

	NHT(+)*	NHT(−)
routine H-E No. pts (%)	4 (14.8)	4 (26.7)
step section & IHC No. pts (%)	5 (18.5)	0 (0)
total LN mets No. pts (%)	9 (33.3)	4 (26.7)

* NHT: neoadjuvant hormone therapy, Figures in parentheses are in percentage

## FUTURE PROSPECTS

Based on the report of Wawroschek *et al*., and our own results, if the SN is metastasis-free the patient can be deemed to be more likely negative for LN metastases. In this way, the SN concept can be considered to be valid in prostate cancer as well. In contrast, when metastases are present in the SN, it is thought that not all LN metastases can be resected with SN biopsy alone since in numerous cases affected LN will be located beyond the range of standard dissection, and moreover, metastases may be present in non-SN as well. However, all hitherto conducted research on SN in prostate cancer has been investigative in nature and undertaken at single institutions. In future, to promote research in this field and to aim for widespread clinical application various problems will first have to be overcome. First, multiinstitutional studies will be needed, and at a minimum there will have to be standardization of various influencing factors such as the type of tracer used, length of the period from tracer administration to SN biopsy, and extent of the dissection for the sake of verification. In future, in addition to technical improvements and accumulation of experience, the planning of well-designed collaborative studies and randomized comparative trials will be needed to achieve further advances in research on SN in prostate cancer.

The primary goal of SN research is to be able to make available to patients the least invasive surgical intervention possible. In future, it would be desirable to realize SNNS with a less invasive method. A representative low-invasive method currently applicable is laparoscopic surgery. Our group has adopted endoscopy-assisted minilaparotomy, in which a small 5cm lower abdominal midline incision is made, through which all surgical tools including the endoscope are inserted and SN biopsy and RPx performed.[35] To perform SN biopsy through this small hole, it is useful to obtain detailed information beforehand about the anatomical position of the SN from the above-mentioned SPECT-CT fusion image. For SNNS to be widely applied clinically many research issues remain to be addressed, but various technical problems have already been overcome and have facilitated the adoption of less invasive surgical methods. Further advances in research are awaited in the near future.
